# Atypical posterior reversible encephalopathy syndrome: A lentiform fork sign following transplantation

**DOI:** 10.1002/ccr3.9100

**Published:** 2024-07-31

**Authors:** Franco E. Appiani, Carlos S. Claverie, Francisco R. Klein

**Affiliations:** ^1^ Universitat Internacional de Catalunya Barcelona Spain; ^2^ Neurology Department Favaloro University Hospital Buenos Aires Argentina; ^3^ Neurology Department Hospital CIMA Sanitas Barcelona Spain; ^4^ Stroke Center Favaloro University Hospital Buenos Aires Argentina; ^5^ Faculty of Medical Sciences Favaloro University Buenos Aires Argentina; ^6^ Critical Care Department Favaloro University Hospital Buenos Aires Argentina

**Keywords:** brain edema, immunosuppressive drugs (ISDs), lentiform fork sign, reversible posterior encephalopathy syndrome (PRES), seizures, transplant recipient

## Abstract

**Key Clinical Message:**

Posterior Reversible Encephalopathy Syndrome, typically characterized by parieto‐occipital vasogenic edema, can present atypically, as a bilateral symmetrical vasogenic edema in the basal ganglia, featuring the called “lentiform fork sign.” Prompt recognition of such variations is crucial for accurate diagnosis and tailored management, highlighting the complexity of this syndrome's manifestations.

**Abstract:**

Posterior Reversible Encephalopathy Syndrome (PRES) manifests as transient neurological symptoms and cerebral edema, commonly associated with immunosuppressive drugs (ISDs) in transplant recipients. ISDs can lead to endothelial dysfunction and compromise the blood–brain barrier. Typically, PRES exhibits identifiable MRI patterns, often demonstrating vasogenic edema in the bilateral parieto‐occipital white matter. Identifying unique presentations, such as the recently observed “lentiform fork sign,” commonly seen in uremic encephalopathy, emphasizes this syndrome's broad spectrum manifestations. A 19‐year‐old male, who underwent bilateral lung and liver transplantation, experienced a bilateral tonic–clonic seizure of unknown onset 47 days post‐surgery. MRI findings revealed an unconventional PRES pattern, featuring the “lentiform fork sign” as bilateral symmetrical vasogenic edema in the basal ganglia, surrounded by a hyperintense rim outlining the lentiform nucleus bilaterally. Subsequent management, including ISD modification and magnesium supplementation, resulted in clinical and neuroimaging resolution. An almost complete clinical and radiological resolution was achieved after 14 days. The occurrence of PRES in transplant recipients highlights the intricate interplay among ISDs, physiological factors, and cerebrovascular dynamics, potentially involving direct neurovascular endothelial toxicity and disruption of the blood–brain barrier. Neuroimaging plays a pivotal role in diagnosis. The distinctive “lentiform fork sign” was observed in this patient despite the absence of typical metabolic disturbances. Management strategies usually involve reducing hypertension, discontinuing ISDs, correcting electrolyte imbalances, and initiating antiseizure drugs if necessary. Identifying the presence of the “lentiform fork sign” alongside typical PRES edema in a patient lacking renal failure emphasizes that this manifestation is not solely indicative of uremic encephalopathy. Instead, it might represent the final common pathway resulting from alterations in the blood–brain barrier integrity within the deep white matter. Understanding such atypical imaging manifestations could significantly aid earlier and more precise diagnosis, influencing appropriate management decisions.

## Graphical abstract

Atypical posterior reversible encephalopathy syndrome (PRES) in transplant recipients. This study describes a case of PRES in a 19‐year‐old male following bilateral lung and liver transplantation. The patient presented with a bilateral tonic‐clonic seizure and exhibited an unconventional MRI pattern known as the “lentiform fork sign,” indicating bilateral symmetrical vasogenic edema in the basal ganglia. Management involving immunosuppressive drug modification and magnesium supplementation led to a significant clinical and neuroimaging resolution within 14 days. This case underscores the diverse manifestations of PRES, the pivotal role of neuroimaging in diagnosis, and the need for tailored management strategies in transplant recipients.

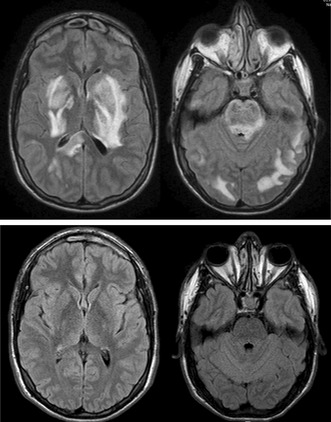

## 1 INTRODUCTION

The posterior reversible encephalopathy syndrome (PRES) presents as focal neurological symptoms associated with neuroimaging of brain edema, frequently evolving toward remission. It typically manifests as a transient episode of encephalopathy, seizures, and visual disturbances within specific patient populations. Originally described in patients with autoimmune conditions, it also occurs in solid organ transplant (SOT) recipients, possibly due to the effect of immunosuppressive drugs (ISDs).^1^ In these conditions, presumed damage to the blood–brain barrier results from endothelial dysfunction caused by exposure to the toxic effects of these agents, or is precipitated by cerebrovascular dysregulation following acute hypertension.^2^

Common brain magnetic resonance image (MRI) patterns associated with PRES include vasogenic brain edema in the white matter, with a dominant parieto‐occipital pattern, bilateral watershed pattern, or superior frontal sulcus pattern. However, atypical localizations such as frontal, temporal, basal ganglia, brainstem, or cerebellar involvement can also occur.^3^ Recently, a bilateral occurrence of basal vasogenic brain edema outlining the lentiform nucleus, known as the “lentiform fork sign” (typically described as a reliable sign of uremic encephalopathy), has been identified in patients experiencing PRES without metabolic disturbances.^4^, ^5^, ^6^, ^7^

In this report, we present a case of a recent bilateral lung and liver transplant recipient with a diagnosis of cystic fibrosis who experienced his first‐ever seizure episode, reflecting PRES with an atypical neuroimaging pattern, including the lentiform fork sign.

## 2 CASE HISTORY

A 19‐year‐old male patient presented to the Emergency Department with an unclear onset seizure progressing to a bilateral tonic–clonic episode lasting approximately 1 mi, followed by an extended postictal state. His medical history included recent bilateral lung and liver transplantation (47th day) due to cystic fibrosis and cirrhotic liver disease, without complications. Notably, the family reported a 48‐h history of headache, nausea, and vomiting preceding the seizure. Upon admission, he was receiving ISDs (tacrolimus), antibiotics, and prophylactic antivirals.

## 3 DIFFERENTIAL DIAGNOSIS, INVESTIGATIONS, AND TREATMENT

**FIGURE 1:**
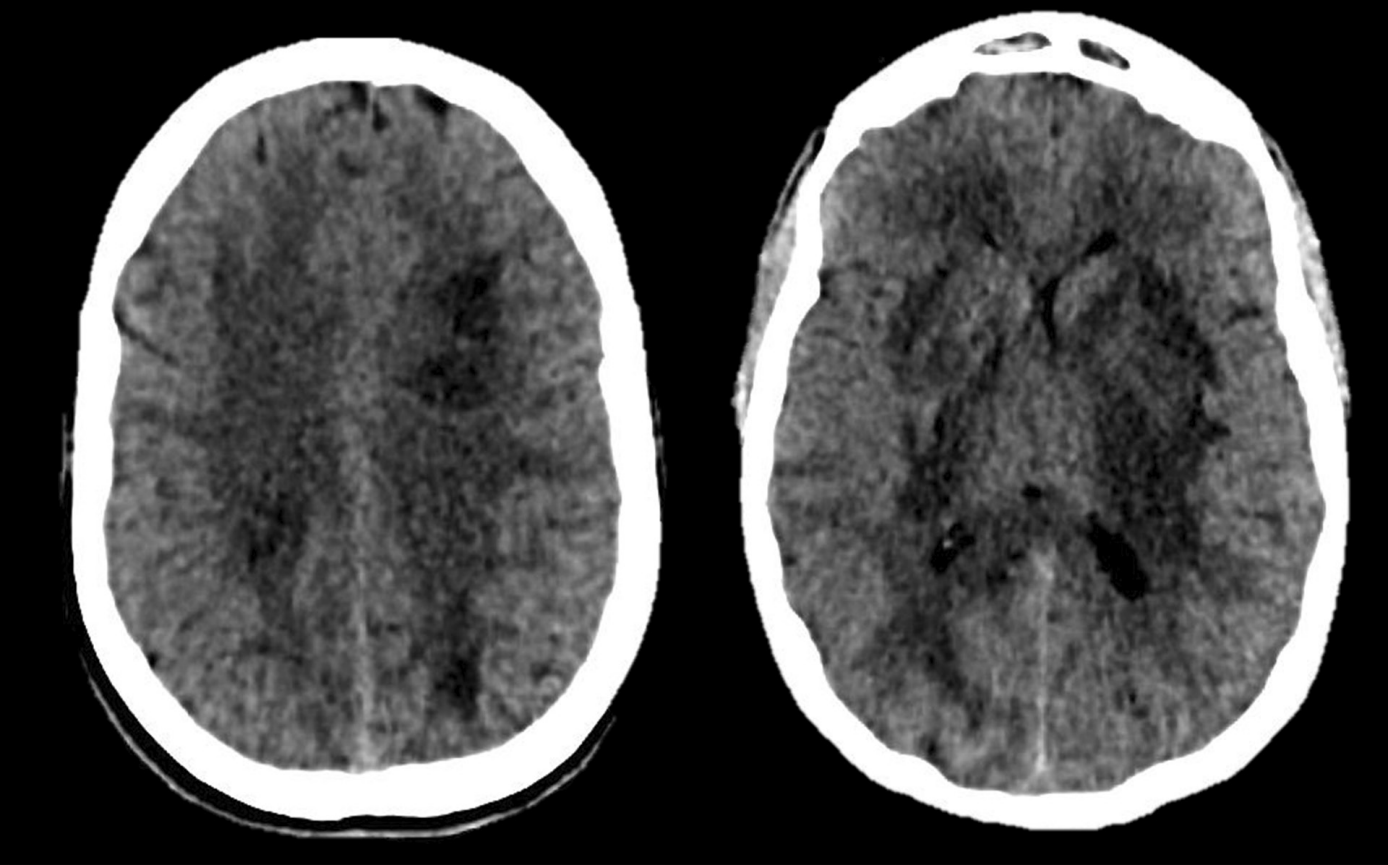
Computed Axial Tomography of the skull showing extensive bilateral subcortical basal and periventricular hypodensity with extension into the diencephalic and pontine‐medulla junction regions, along with a mild mass effect on the frontal horn of the lateral ventricle.

**FIGURE 2:**
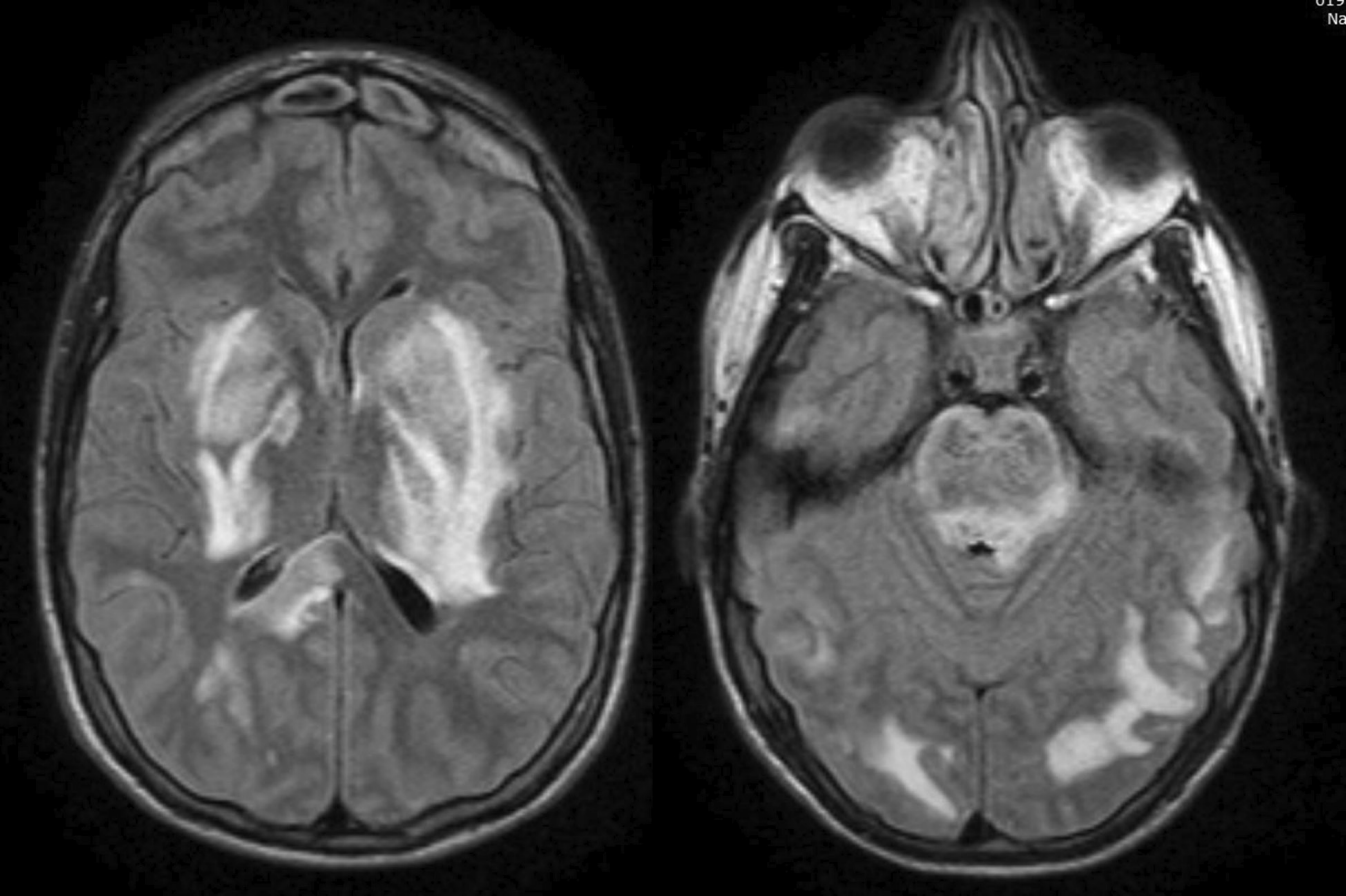
Brain Magnetic Resonance Imaging revealing extensive hyperintense lesions in T2 and FLAIR sequences, hypointense in T1, involving bilateral subcortical and periventricular posterior regions, with pontine medulla junction involvement compatible with vasogenic edema. The hyperintense rim that delimits the lentiform nucleus suggests the lentiform fork sign. No diffusion restriction was observed.

Initial postictal neurological examination revealed somnolence, right hemispheric myotatic hyperreflexia, hypoesthesia, and paraesthesia. Laboratory findings showed leucocytosis (26.7 × 10^3^/mm^3^), mild hyponatremia (Na 130 mEq/L), severe hypomagnesemia (0.8 mg/dL), and elevated tacrolimus levels (14 mg/dL), with mild liver dysfunction (AST 59 U/L; ALT 88 U/L) and normal renal function (Cr 0.9 mg/dL, Urea 41 mg/dL). Venous and arterial blood gases immediately after magnesium correction were unremarkable (Venous: pH 7.42, PaCO_2_ 33 mmHg, PaO_2_ 42 mmHg, CO3H 21 mEq/L, EB −2.9, SatO_2_ 77%. Arterial: pH 7.45, PaCO_2_ 33 mmHg, PaO_2_ 121 mmHg, CO_3_H 22.6 mEq/L, EB −0.9, SatO_2_ 98%). On the same day, several complementary studies were performed. Electroencephalogram (EEG) displayed a normal background rhythm (8–10 Hz) without epileptiform activity. A brain computed tomography scan (CT) revealed extensive bilateral subcortical hypodense lesions (Figure 1). Lumbar puncture showed normal pressure and cell count with an elevated protein level (1.76 g/L). Viral PCRs (Epstein Barr, Enterovirus, Herpes Simplex 1, 2, and 6, Varicella Zoster), Gram stain, India ink, Kinyoun staining, Cryptococcus, and JC antigen tests, as well as cultures for common, atypical, and fungal pathogens were performed, showing negative results. MRI displayed extensive bilateral symmetrical vasogenic brain edema in basal ganglia and bilateral subcortical areas, involving the diencephalon and pontine‐medulla junction, with a recognizable lentiform fork sign (Figure 2). Methanol or ethylene glycol intoxication, abnormal neurodevelopment, and familial metabolic disorder symptoms were ruled out through patient history and interrogation.

Therapeutic measures included urgent magnesium replacement, and switching immunosuppressive drugs from tacrolimus to cyclosporine. Antiseizure treatment with levetiracetam was initiated on the same day during the extended postictal state due to suspicion of status epilepticus.

## 4 OUTCOME AND FOLLOW UP

**FIGURE 3:**
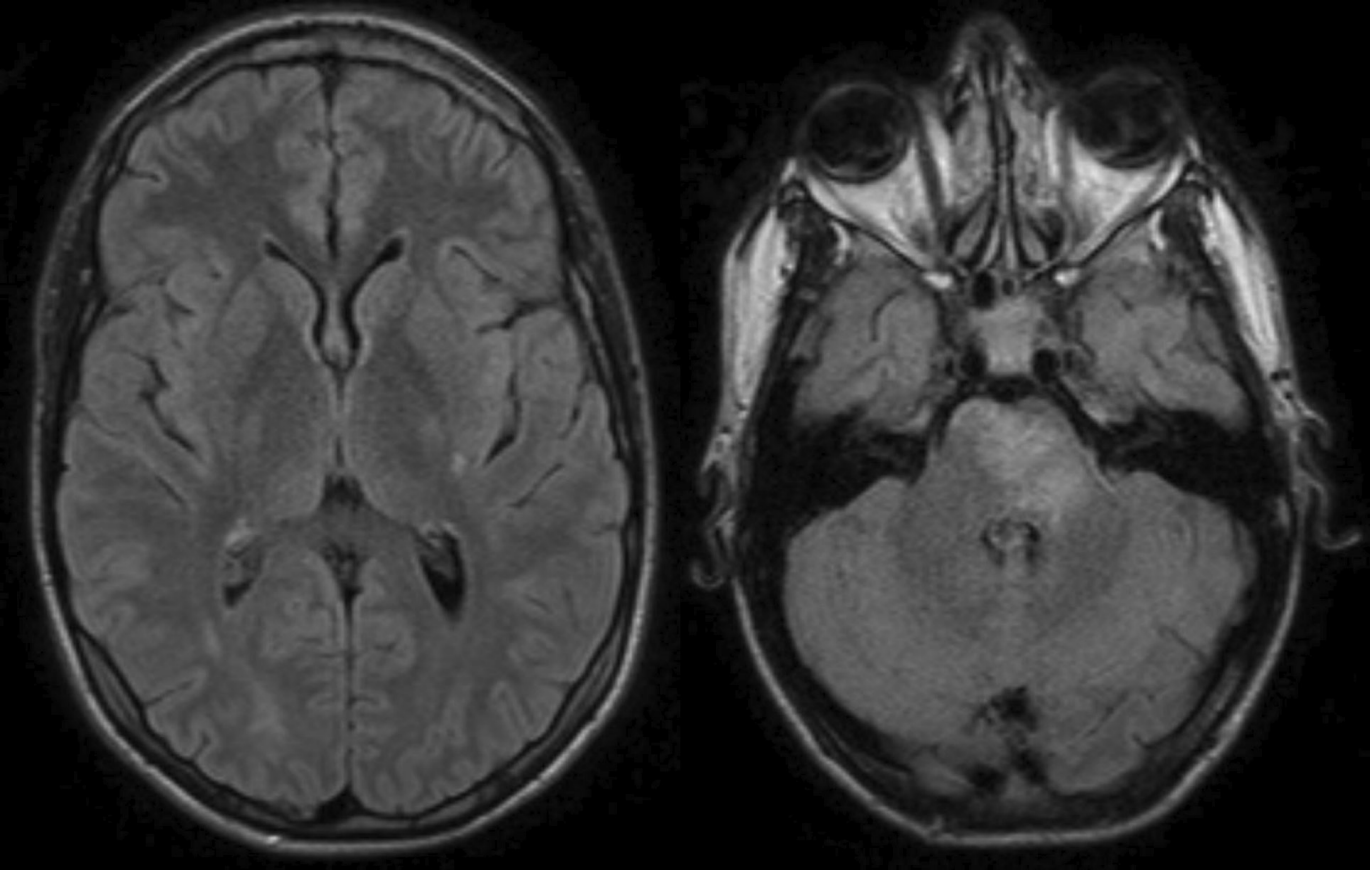
Brain Magnetic Resonance Imaging displaying a hyperintense lesion in the protuberance, the posterior limb of the left internal capsule, and the homolateral fronto‐insular subcortical region in T2 and FLAIR sequences, compatible with vasogenic edema in resolution.

**FIGURE 4:**
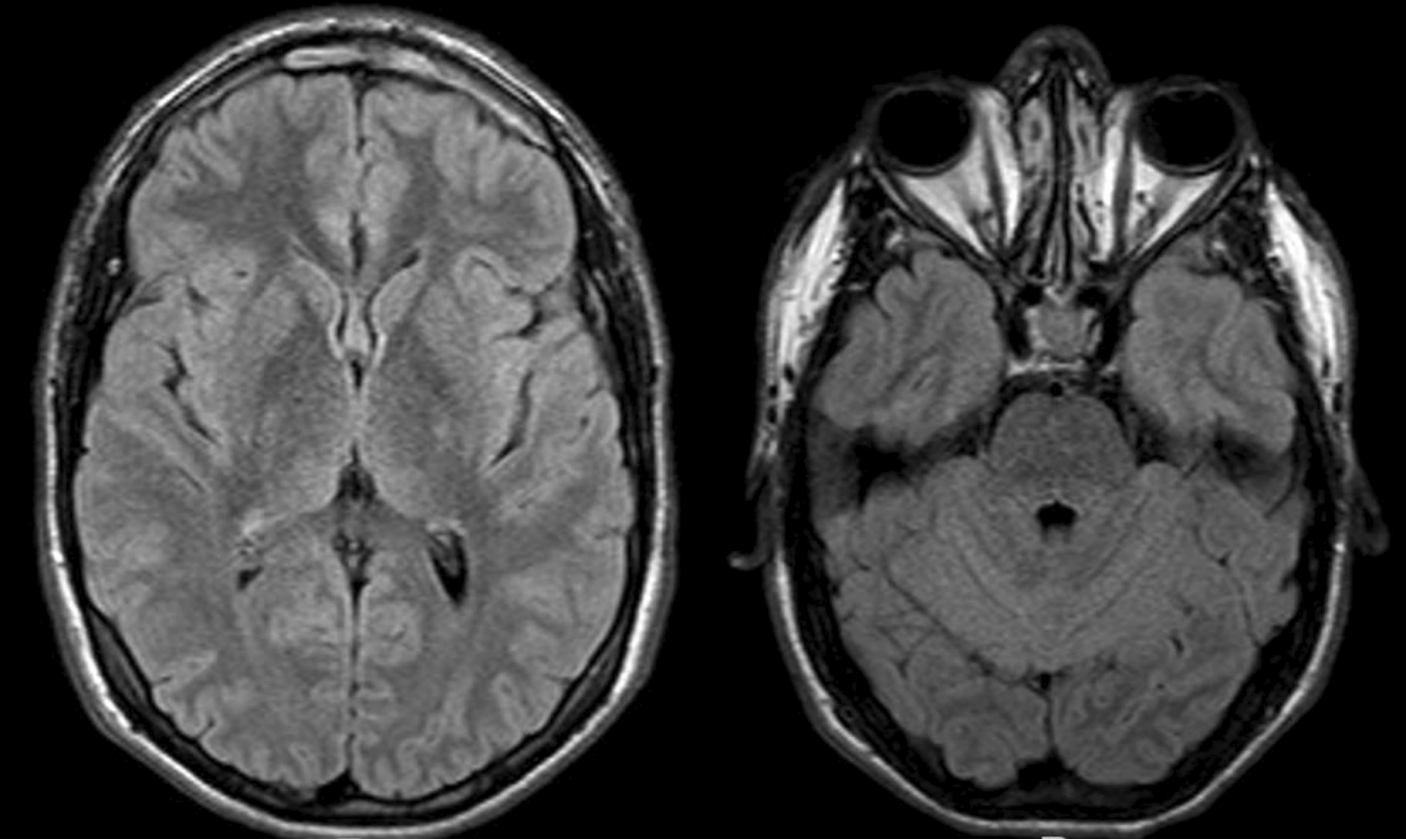
Normal Brain Magnetic Resonance Imaging of the same patient, with no vasogenic edema or sequelae.

By Day 2, the patient became asymptomatic, indicating improvement in neurological condition, with no recurrence of epileptic seizures and subsequent normal EEG results (three in the following days). Before discharge, 14 days after the first assessment, a follow‐up brain MRI was performed, revealing an almost complete resolution of the previously described findings (Figure 3). After discharge, the patient remained seizure‐free, with a normal brain MRI (Figure 4) and EEG results performed 2 years after the initial episode. In this context, the decision was made to discontinue his antiseizure medication.

Given the clinical presentation and neuroimaging findings, the case was interpreted as an atypical PRES in a liver and lung transplant recipient, in the context of elevated tacrolimus plasma concentrations associated with hypomagnesemia. The patient achieved clinical and radiological resolution, with no further complications over 4 years of follow‐up by now.

## 5 DISCUSSION

PRES in transplant recipients reveals complex interactions between ISDs, physiological factors, and cerebrovascular dynamics, potentially causing direct neurovascular endothelial toxicity and blood–brain barrier disruption. PRES occurs in 1% of adult liver transplant and 2% of lung transplant recipients, with seizures as the most common clinical presentation.^1^, ^8^ The patient's regimen of ISD usually plays a role, especially when it involves tacrolimus (calcineurin inhibitors), which is recognized as a frequent culprit in PRES development and seizure triggers. These toxic effects may arise due to systemic hypertension and also as a direct neurovascular endothelial toxicity effect. Moreover, as found in our patient, tacrolimus has a significant effect on urinary magnesium wasting, possibly leading to hypertension and encephalopathy after transplantation.^9^, ^10^

The lentiform fork sign denotes bilateral symmetrical vasogenic brain edema in the basal ganglia, enclosed by a hyperintense rim in T2‐weighted images (T2‐WI) and fluid‐attenuated inversion recovery (FLAIR) sequences. It is commonly observed in metabolic disturbances, such as uremic encephalopathy, diabetes mellitus, and methanol or ethylene glycol intoxications. Neuroimaging played a crucial role in this case without those metabolic alterations, with CT and MRI scans revealing this characteristic sign in typical PRES edema. As recently described in the literature, neither uremia nor metabolic acidosis was observed in this case.^4^, ^6^ As implicit in the acronym PRES, the findings associated are typically reversible, showing a return to normal clinical and imaging results once the underlying cause is addressed and corrected. However, in certain instances, regions with limited diffusion can lead to lasting damage to the brain tissue.^11^ In our case, the patient exhibited vasogenic brain edema in an infrequent distribution without restricted diffusion or microbleeds. The patient underwent rapid and substantial almost complete recovery within 2 weeks, followed by complete resolution before 14 days of in‐hospital follow‐up.

The acute management of PRES involves supportive measures, primarily addressing and correcting the underlying causes, including a gradual reduction of acute hypertension, discontinuation, and/or switching ISDs, with treatment of the eventual electrolyte imbalances.^12^ Randomized trials for treatment options are lacking, and guidelines are based on consensus. In our case, antiseizure drug therapy was initiated in the acute phase due to the suspicion of status epilepticus and continued due to the high risk of recurrence. It was discontinued once the brain edema resolved and the patient had a long seizure‐free period with normal EEGs controls. Long‐term antiseizure medication is usually unnecessary for most patients post‐PRES, with discontinuation considered after the acute phase.

Our understanding suggests that distinctive vasogenic brain edema affecting the basal ganglia, referred to as the lentiform fork sign, does not stem from a singular metabolic origin but might represent a final common pathway resulting from compromised blood–brain barrier integrity in the deep white matter.

Our case suggests that the lentiform fork sign, previously associated with uremic encephalopathy, can occur without typical metabolic disturbances, indicating a broader spectrum of PRES presentations. Prompt recognition of such atypical features is essential for accurate diagnosis and optimal management.

Ongoing research and consensus‐based guidelines are vital for enhancing our understanding and management of PRES in transplant recipients, ensuring timely intervention, better outcomes, and minimizing long‐term neurological consequences.

The author has obtained written informed consent from the patient before submission.

## AUTHOR CONTRIBUTIONS

**Franco E. Appiani:** Conceptualization; data curation; formal analysis; investigation; methodology; project administration; resources; software; supervision; validation; visualization; writing – original draft; writing – review and editing. **Carlos S. Claverie:** Formal analysis; investigation; methodology; project administration; resources; supervision; validation; writing – review and editing. **Francisco R. Klein:** Conceptualization; formal analysis; investigation; methodology; project administration; resources; supervision; validation; visualization; writing – review and editing.

## FUNDING INFORMATION

No financial disclosures or funding to report.

## CONFLICT OF INTEREST STATEMENT

The authors declare no conflicts of interest.

## ETHICS STATEMENT

The manuscript follows the Declaration of Helsinki principles.

## CONSENT

Written informed consent was obtained from the patient to publish this report in accordance with the journal's patient consent policy.

## Data Availability

The data that support the findings of this study are available on request from the corresponding author. The data are not publicly available due to privacy or ethical restrictions.
